# Preparation and *in vitro* evaluation of injectable formulations of levothyroxine sodium using *in situ* forming hydrogel temperature-responsive systems based on PLA-PEG-PLA and PLGA-PEG-PLGA triblock copolymers

**DOI:** 10.22038/IJBMS.2022.62576.13842

**Published:** 2022-03

**Authors:** Jebraeil Movaffagh, Farzin Hadizadeh, Elham Khodaverdi, Bahnaz Khalili, Seyedeh Nesa Rezaeian Shiadeh, Hossein Kamali, Fatemeh Oroojalian

**Affiliations:** 1 Department of Pharmaceutics, School of Pharmacy, Mashhad University of Medical Sciences, Mashhad, Iran; 2 Targeted Drug Delivery Research Center, Pharmaceutical Technology Institute, Mashhad University of Medical Sciences, Mashhad, Iran; 3 Department of Medicinal Chemistry, School of Pharmacy, Mashhad University of Medical Sciences, Mashhad, Iran; 4 Biotechnology Research Center, Pharmaceutical Technology Institute, Mashhad University of Medical Sciences, Mashhad, Iran; 5 Department of Advanced Technologies, School of Medicine, North Khorasan University of Medical Sciences, Bojnurd, Iran; # These authors contributed equally to this work

**Keywords:** In situ forming hydrogel- gelling, Levothyroxine sodium, Smart hydrogels, Temperature-responsive- systems, Triblock copolymer

## Abstract

**Objective(s):**

Recently, great attention has been paid to developing new drug delivery systems to manage the rate, time, and site of drug release. We aimed to design a novel drug delivery system to support targeted and gradual delivery of levothyroxine sodium.

**Materials and Methods:**

The triblock copolymers of PLA-PEG-PLA and PLGA-PEG-PLGA were constructed using the ring-opening copolymerization method and then purified and characterized by 1H-NMR, DSC, and GPC techniques. The phase transition temperature of the polymers was determined, and levothyroxine sodium stability was investigated in a phosphate-based buffer (pH 7.4). *In vitro* drug release into the PBS was measured at different concentrations of the triblocks for one month.

**Results:**

The results of NMR and GPC showed successful fabrication of the copolymers with low molecular weight dispersion and T_g_ points of -8.19 ^°^C and -5.19 ^°^C for PLA-PEG-PLA and PLGA-PEG-PLGA, respectively. Stability tests showed that during one month, most of the triblocks’ masses degraded at 37 ^°^C while levothyroxine sodium remained stable. Initial burst release of the drug in both copolymers is inversely correlated with the concentration of the polymer. Evaluation of drug release for 35 days showed that PLA-PEG-PLA had a slower drug release rate than PLGA-PEG-PLGA.

**Conclusion:**

Considering the low initial burst release, as well as continuous and long-term release kinetics of PLA-PEG-PLA and PLGA-PEG-PLGA copolymers, they can be used to gradually deliver levothyroxine sodium, obviating the need for frequent administrations and concerns over drug-food interactions.

## Introduction

In recent decades, biodegradable hydrogels have acquired great attention because of their ability to protect the structure of unstable molecules such as proteins. These hydrogels have high water content and are hydrophobic intelligent materials that are responsive to various stimuli. The three-dimensional structure of hydrogels confers on them suitable mechanical stability. Furthermore, they are implantable because of their acceptable safety and biocompatibility. Hydrogels, due to considerable water content and high porosity, can enclose and then release large hydrophilic compounds such as peptide-based drugs. Because of these features, hydrogels are used by scientists for designing slow-release drug delivery systems.

Intelligent hydrogels (i.e., responsive to stimuli such as environmental factors) respond to natural stimuli by modifying their structural and physical properties, and permeability. These stimuli can be divided into three main categories: physical (heat, magnetic and electric fields, light, pressure, sound, etc.), chemical (pH, glucose, oxidants, etc.), and biological (antigens, ligands, enzymes, etc.) (1-5).

Temperature-responsive hydrogels are known for their ability to swell and shrink depending on temperature variations (6). In fact, heat is a common stimulus used to manipulate intelligent hydrogels (7). Poloxamer copolymers (POE-PPO-POE) have been extensively studied due to their reversible temperature-sensitive properties (8). Another temperature-responsive hydrogel is the PLGA-PEG-PLGA triblock copolymer that has been widely studied due to its interesting advantages such as easy synthesis, tunable gel performance, as well as high and controllable degradability (9, 10).

Stimulus-responsive *in situ* gel-forming hydrogels, which are actually aqueous mixtures of gel precursors and low-viscose biologically active materials, undergo gelation in response to changes in factors such as temperature, pH, and the type of the solvent when they are injected into the body. One of the most important advantages of these hydrogels is that they do not require surgery to be delivered to the body (in contrast with implantable hydrogels). Besides, stimulus-responsive hydrogels can be loaded with different types of drugs (1, 7, 8).

Based on the arrangement of their constituents, segmented-block copolymers are divided into several types (11). Polylactic acid (PLA) and poly (lactic-co-glycolic acid) (PLGA) are two biodegradable and biocompatible polymers widely used in biomedical sciences (12). Macromolecules such as proteins, peptides, genes, vaccines, antigens, and human growth factors have already been successfully packaged into PLGA nanoparticles or microparticles. Polylactic acid polymers are metabolized into lactic acid monomers in the body, and PLA-based nanoparticles and microparticles have been used as controlled-release systems in recent years to enclose various types of drugs (13).

Levothyroxine sodium is a synthetic form of T4 hormone, which is biochemically and physiologically recognizable from the body’s natural hormone and is prescribed in quantitative or qualitative deficiencies of T4 (14). The oral form of levothyroxine sodium is prescribed for treating primary (thyroid), secondary (pituitary), and tertiary (hypothalamic) hypothyroidism (15). It is also used to treat euthyroid goiters such as thyroid nodules, subacute or chronic lymphocytic thyroiditis, multinodular goiter, and thyroid cancer. The drug is also utilized after thyroidectomy and as a complementary agent following surgery and radiotherapy (16). Levothyroxine sodium is mainly absorbed in the small intestine, specifically in the duodenum, jejunum, and ileum, and to a lesser extent, in the stomach (17-19). In people with normal thyroid function (i.e., euthyroid), the maximum concentration (T_max_) of the drug is reached in about two hours after administration, while in those with hypothyroidism, this time is close to three hours, which can even be extended by consumption of some foods (18). In individuals with normal thyroid function, the bioavailability of levothyroxine sodium is about 60–80%; however, this rate is slightly higher in those with hypothyroidism or hyperthyroidism. The bioavailability of levothyroxine is lower in the presence of food, and its uptake is also affected by gastric pH (20, 21). Despite the apparent high solubility of levothyroxine sodium in aqueous solvents, the hormone contains an alanine residue that delays its intestinal uptake (22). The serum level of the drug remains high from 2–4 hr to up to six hours after oral consumption, necessitating re-administration (23). The drug’s uptake is affected by gastrointestinal health and the presence of foods and caffeine (24). The bioactivity of the oral forms of the drug varies considerably (from 40 to 80%), compromising their biological efficiency (2).

In patients with hypothyroidism, levothyroxine sodium is used in fasting, which needs repeated daily administration, making patients forget on-time drug usage that may exaggerate hypothyroidism complications. In addition, the serum concentration of the drug declines during the day, reducing its efficacy. Accordingly, slow-release formulations of the drug have been suggested to overcome problems with its bioavailability and patient compliance. 

Recently, PLGA-containing solid-phase *in situ* forming implants (ISFI) have been developed as slow-release drug delivery systems. These systems also contain N-methyl pyrrolidone (NMP), which is frequently used as a biocompatible water-miscible organic solvent. In aqueous milieu, NMP and water start to diffuse into the release medium and the polymer matrix, respectively (i.e., the phase inversion phenomenon). The contact of water with the matrix causes the polymer to precipitate, resulting in solid polymeric implant formation (25). Currently, a number of commercial compounds use NMP in their structure, including Eligard^®^ (PLGA-based leuprorelin acetate), Doxirobe^®^ (PLA-based doxycycline hyclate), and Nuflor^®^ (PEG-based Florfenicol) (26). A disadvantage of ISFIs is that they deliver a high rate of initial release (i.e., from 15 to 80% of the drug’s content during the first 24 hr). Another concern is that the drug released in the initial burst phase, due to NMP solvent burst release, may cause local inflammation and systemic toxicity (27). The use of the NMP solvent, allowing for easy dissolving of the drug, and immediate gelation at the injection site, owing to the nature of the polymer, are two main benefits of this sustained-release drug formulation. It has also been noted that the hydrogen bonding of PEG (a component of the triblock) with NMP and the thermal features of the triblock prevent the rapid diffusion of the solvent into the release medium. The mixture containing the polymer and the NMP solvent is poured into one syringe, which is then mixed with the drug Power to prepare ISFI formulations. After the injection of this formulation, gelation occurs at the injection site. Two mechanisms have been proposed for gel formation: the thermal response of the polymer and the phase inversion phenomenon, solvent-induced phase inversion (28).

The use of the PLGA-PEG-PLGA triblock instead of PLGA alone for preparing levothyroxine sodium formulations has been suggested to lower the initial drug release rate, as reported by Kamali *et al.* who investigated the drug release *in vitro* for three weeks (29). The utilized PLGA-PEG-PLGA triblock also offers the advantage of being liquid at room temperature and immediate gelation at 37 °C (25, 29-31), obviating the need for surgical implantation of this injectable triblock. On the other hand, because of high instability of the PLGA-PEG-PLGA triblock in exposure to water, it cannot be used for drugs that need water for their preparation. As an alternative, the NMP solvent can be utilized to address this problem. 

**Scheme 1:**
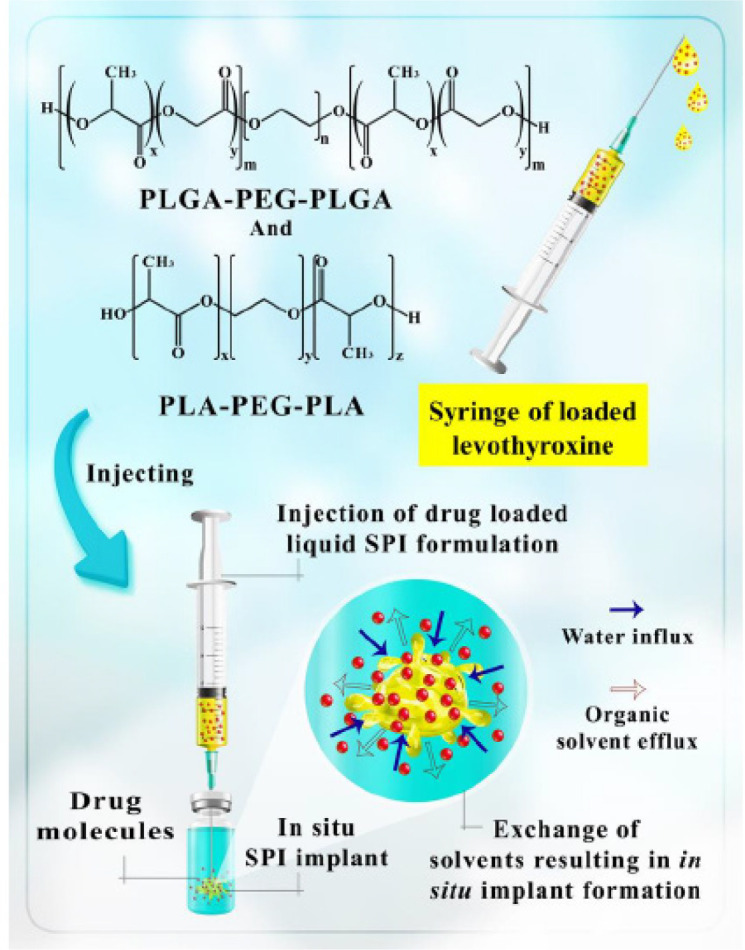
An overview of formulation of PLGA-PEG-PLGA or PLA-PEG-PLA *in situ* gel formation with NMP

In this study, we intended to develop an *in situ* controlled drug-release system for levothyroxine sodium using two injectable, biodegradable, and biocompatible triblock copolymers (PLA-PEG-PLA and PLGA-PEG-PLGA). Due to presence of glycolide in its structure, PLGA-PEG-PLGA is more hydrophilic than PLA-PEG-PLA, which is thought to affect the release of levothyroxine sodium. Actually, the hydrophilicity effects of PLGA and PLA blocks on the rate of the release process of sodium levothyroxine from the NMP containing gel system have been investigated. In addition, drug release patterns, drug stability, and other features of PLA-PEG-PLA and PLGA-PEG-PLGA triblock co-polymer systems were compared (Scheme 1). 

## Materials and Methods

***Materials***

Lactide, glycolide, PEG (Mw=1500 Da), N-Methyl-2-pyrrolidone, and 3- (4,5-dimethylthiazol-2-yl) - 2,5-diphenyltetrazolium bromide (MTT) were obtained from Sigma-Aldrich. Levothyroxine sodium was purchased from Iran Hormone Co. (Iran). Cell culture medium (RPMI1640), fetal bovine serum (FBS), penicillin-streptomycin, and trypsin were purchased from Gibco (Germany). Vivitrol® was obtained from Alkermes (USA), and other materials were from Merck (Germany).

***Methods***

*Synthesis of triblock copolymers*

**Figure 1:**
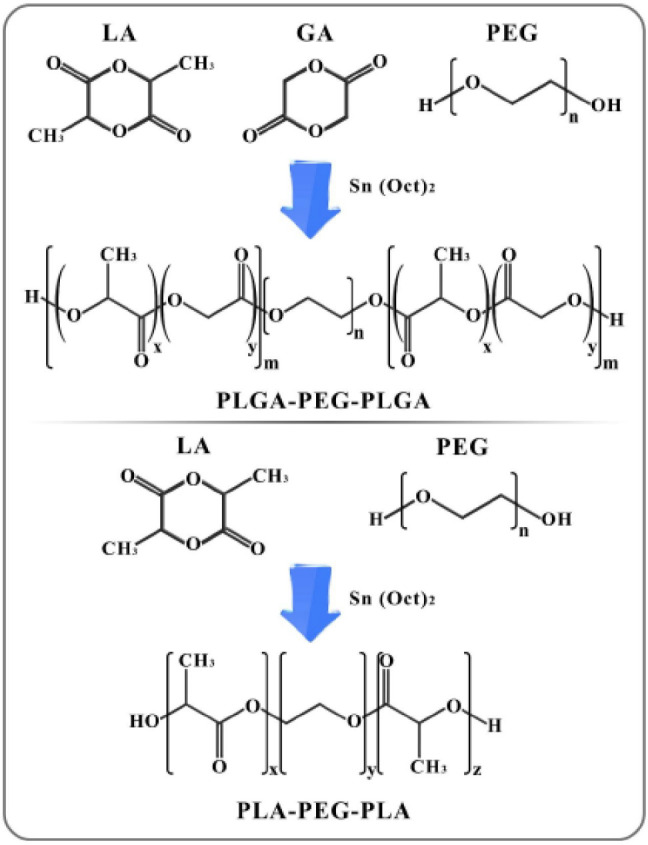
Synthesis of PLGA-PEG-PLGA or PLA-PEG-PLA triblock polymer via ring-opening polymerization

**Polymer fabrication in microwave**: PLA-PEG-PLA and PLGA-PEG-PLGA were synthesized using the ring-opening polymerization (ROP) method. Initially, to dehydrate PEG, the polymer was placed into a microwave for 10 min (100 watts). Then lactide (for synthesis of PLA-PEG-PLA) or the combination of glycolide: lactide (i.e., the PLGA polymer, in the molar ratio of 7: 1, respectively, for synthesis of PLGA-PEG-PLGA) was added to a balloon containing PEG (the mass (g) ratio of 10: 3 for the middle polymer (PEG) and the side polymers, respectively). The mixture was melted at 130 ^°^C. Finally, 10 μl of pure Sn (Oct)_2_ catalyst was added to the reaction, and incubation continued for 10 min at 130 ^°^C. The general scheme of the synthesis process of the triblock polymers (PLGA-PEG-PLGA and PLA-PEG-PLA) has been shown in Figure 1.

*Triblock copolymer purification*

Purification was performed by dissolving the copolymers (PLA-PEG-PLA or PLGA-PEG-PLGA) in cold water and then precipitating impurities by immersing them in hot water. Twenty-four hours after fabrication, the synthesized copolymers were immersed into deionized water at 37 ^°^C, and then the copolymer container was placed on ice (4 ^°^C) to generate the sol phase. In this condition, unreacted materials and the copolymer would be dissolved. Next, the temperature was elevated up to 65 ^°^C to allow the fabricated polymers to precipitate, while unreacted materials remained dissolved in the supernatant. The supernatant was then discarded. These steps (i.e., the addition of deionized water, lowering the temperature, and then increasing the temperature) were repeated three times to purify the polymer. After purification, lyophilization was performed to completely remove the water. The polymers fabricated were initially placed in a freezer at -20 ^°^C for 24 hr and then transferred into a freeze-dryer (10). 

*Fabrication efficiency calculation*

For this purpose, the weight of the dried product was divided by the total mass of the materials used at the onset of the reaction. 

*Triblock polymer identification and characterization*

^1^ *H-NMR*

To identify the copolymers, the presence of lactide, PEG, and glycolide subunits (for the PLGA-PEG-PLGA triblock copolymer) was investigated via 1H-NMR spectroscopy. First, the samples were dissolved in deuterated chloroform, and then their spectra were determined at 300 MHz. To determine their characteristics, the obtained spectra were compared with a reference (32). 

*Molecular weight determination by gel permeation chromatography*

The distribution of molecular weight (Mw) and molecular mass of the fabricated triblock polymers were determined by Gel Permeation Chromatography (GPC) using the tetrahydrofuran solvent at a flow rate of 1 ml per minute. Polystyrene was used to calibrate the system before sample injection, and a solvent was used to wash out the sample injected inside the separating column.

*Determination of phase change temperature*

To determine the phase change temperature (i.e., the gelation point), the tube inversion method was used. First, 10-50% concentrations of the copolymer’s aqueous solution were prepared in a PBS buffer (pH 7.4) and incubated in the refrigerator for 24 hr, while stirring gently via a magnet until the polymer was completely dissolved. Then a certain volume of each sample was transferred to similar tubes that were placed in a water bath with the temperature gradually increasing from 25 ^°^C to 55 ^°^C (0.5 ^°^C per minute). After each step, to reach a balanced temperature, a 2-minute lag phase was considered. Following each temperature rise/stop phase, the sample-containing tubes were completely inverted to inspect gel formation (evidenced by the sample remaining attached to the bottom of the inverted tube). The gelation temperature was recorded for each sample. Then the temperature was increased in the same manner until the polymer precipitated in the tube, forming a two-phase system. The temperature at which the sample turned into a two-phase compound was also recorded as the polymer’s precipitation point. The experiment was repeated three times for each sample.

*DSC analysis*

DSC analysis was performed for both PLGA-PEG-PLGA and PLA-PEG-PLA triblock polymers. For this purpose, 3 mg of the samples were placed into special aluminum crucibles, and the thermal behaviors of the samples were within the range of -60 ^°^C to +60 ^°^C (10 ^°^C rise per minute) and were recorded under nitrogen gas flow versus empty aluminum crucibles as the reference.

*Levothyroxine sodium standard curve*

A 10 mg/ml levothyroxine sodium stock solution was prepared by accurately diluting to 1000 ml with PBS, and other standard samples were prepared by making ½ dilutions. Afterward, 50 μl of each sample (in a concentration range of 0.0048 to 10 μg/ml) was injected into the HPLC (in triplicate). Finally, retention time and the area under the peak were recorded. Then the graph of concentration vs the area under the peak was plotted.

*Drug quantification by HPLC*

In this experiment, the mobile phase was the 40%:60% acetonitrile: water solution, to which 0.5 ml of phosphoric acid was added. The Buchner funnel and a vacuum pump were used to separate tiny particles. The flow rate of the mobile phase was 1 ml per minute, and temperature and wavelength were set at 25 ^°^C and 255 nm, respectively. The injection volume was 50 µl, and the infusion was repeated three times. Different concentrations were prepared and the peak absorbance of levothyroxine sodium was determined. At specified times, levothyroxine sodium was measured to determine the peak value. According to the area under the curve and the equation obtained from the graph, the drug’s concentration was determined in each sample.

*Levothyroxine loading in copolymers*

For loading the drug into the polymeric structure, levothyroxine was simply dissolved in the polymer solution. For this purpose, solutions of copolymers were prepared at the 30, 40, and 50% w/w concentrations in the NMP solvent, and then 6 mg levothyroxine sodium was added to each solution and completely dissolved by sonication for 2 hr.

*Viscosity Assessment*

To evaluate the viscosity of the triblock copolymers, 30, 40, and 50 % w/w concentrations of the copolymers were prepared in the NMP solvent. To determine the viscosity of each sample, 3 ml PBS buffer was added to 1 ml of the NMP-polymer mixture, and the samples were incubated for four days at 37 ^°^C. Finally, the supernatant was removed, and the viscosity of the precipitate was measured.

*Drug release examination*

In order to evaluate the drug release rate, 1 ml of each of optimally prepared drug-containing formulations was transferred into a test tube and then placed in a water bath for five minutes to reach 37 ^°^C. Then 10 ml of PBS (37 °C, pH=7.4) was gradually added to the test tube. For investigating gelation, the height of the tube’s constituents was marked; the tips of the tubes were tightly closed, and they were transferred to a shaker water bath (37 ^°^C, speed of 35 rpm) to prevent the formation of a diffusion layer. Subsequently, at specific time intervals (0, 2, 4, 8, 10, and 12 hr, as well as 1, 2, 3, 4, 7, 10, 14, 21, 22, 28, and 35 days), 1 ml of the surface buffer, which contained the drug released, was withdrawn. The volume removed was replaced with 1 ml fresh buffer. Sampling was conducted at longer intervals during the last days because of a decline in the release rate. Finally, levothyroxine sodium level in each sample was determined via HPLC, and a standard graph (cumulative drug release vs time) was prepared.

The percentage of the drug released until t_n_ was calculated according to the following formula (33):

M_n_ = C_n_ V_t_ + ∑ C _(n-1)_ V_s_

M_n_ = True (cumulative) amount of n_th_ sample (μg/ml)

C_n _= Apparent concentration (read) of n_th_ sample (μg/ml)

V_s_ = Volume removed from the sample (ml)

V_t_ = Total volume of the release medium (ml)

∑C _(n-1)_ = Total apparent concentrations (read) of samples between 1^st^ and n-1 (μg/ml)

*Degradation features of triblock copolymers*

To perform the degradation test, weight changes of the synthesized triblock polymers were determined 37 °C after 1, 2, 3, 4, 5, 7, 10, 13, 16, 19, 22, 25, 28, and 31 days. A separate microtube was designated each time for weight measurement. A specific amount of the polymer was added to the microtube, and then the weight of the microtube was measured. The change of the wight of the microtube containing the polymer at the end of the degradation test corresponded to the change of the mass of the polymer. After weighing the microtube at the end of the test, one milliliter of deionized distilled water was added to each sample, and all the microtubes were transferred into a water bath at 37 ^°^C. Each sample was withdrawn at a specified time; the supernatant was gently removed, and the precipitate was kept at room temperature. On day 31^st^ and after the last sample was processed, all the samples were transferred into a freeze-dryer to allow for complete drying before weighing the microtubes. The weight reduction rate reflected polymer degradation, according to which a degradation vs timetable was prepared.

*The effects of the triblock copolymers on the viability of animal cells in vitro*

The MTT assay was used to evaluate the viability of the cells exposed to the triblock copolymers containing levothyroxine sodium. In brief, the L929 mouse fibroblast cell line was purchased from Iran’s Pasteur Institute and cultured in T25 flasks containing RPMI1640 medium supplemented with 10% fetal bovine serum (FBS) and 1% penicillin (100 IU/ml)-streptomycin (100 mg/ml). Cell cultures were placed into an incubator with 5% CO_2_ and 95% humidity at 37 ^°^C until reaching the growth phase. To detach the cells from the bottom of flasks, 0.25% trypsin was used. After two passages, 10,000 cells (at the growth phase) were seeded in the wells of a 96-well plate and incubated overnight. Four wells were assigned to each copolymer formulation. The copolymer was mixed with an equal volume presented to the cells for either 24, 48, or 72 hr. After this step, 10 µl of the MTT solution (5 mg/ml in PBS) was added to each well, and the mixture was incubated for 4 hr at 37 ^°^C. The culture medium was then removed to assess emerged blue-colored sediments. DMSO (100 µl) was added to each well, and the plate was placed on a shaker for five minutes. Finally, the absorption of the resultant solution was read at 570 nm (versus 630 nm as the background). Finally, the viability of the cells was estimated based on the absorption percentages of copolymer-treated cells vs untreated control cells. The experiment was repeated at least three times for each sample, and the results were presented as mean±SD. Relative cell death (R) was calculated using the following formula (34):

R = [(A_test_ / A_control_) × 100] 

In this formula, A_test_ is the absorption of the cells treated with the copolymer, and A_control_ is the absorption of the cells exposed to the culture medium (i.e., the control). Finally, the IC_50_ values of the formulations were calculated using the GraphPad Prism software package.

***Statistical analysis***

Statistical tests (t-test, one-way ANOVA, and two-way ANOVA) were conducted in GraphPad Prism software package.

***Ethical considerations***

This study was approved by the institutional ethical committee (School of Pharmacy, Mashhad University of Medical Sciences, Iran) under the code of IR.MUMS.PHARMACY.REC.1397.001.

## Results

***The synthesis yield of PLGA-PEG-PLGA and PLA-PEG-PLA***

The PLGA-PEG-PLGA (with a lactide to glycolide ratio of 7:1) and PLA-PEG-PLA triblock copolymers were synthesized via the ROP method using a three-neck balloon. The synthesized triblock copolymers appeared light yellow and had semi-solid stickiness and viscosity. The synthesis yields of PLGA-PEG-PLGA and PLA-PEG-PLA were 83.4% ±2.65 and 85.1% ±2.41, respectively. 

***Identification and characterization of triblock copolymers***

^1^ *H-NMR studies*

**Figure 2:**
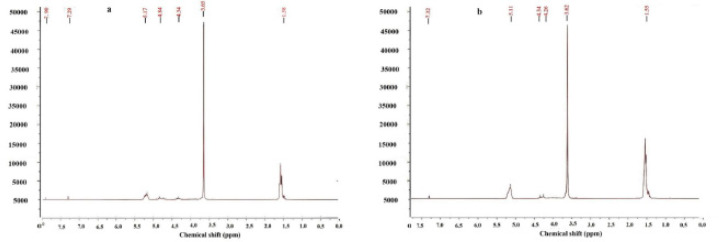
^1^H-NMR spectra of PLGA-PEG-PLGA (a) and PLA-PEG-PLA (b) triblock copolymers

^1^H-NMR was used to determine polymer structure and ratios of lactide (LA) to glycolide (GA), as well as PEG 1500 to PLGA 1500. The spectrum of PLGA 1500-PEG 1500-PLGA1500 has been depicted in Figure 2a. The peaks around 5.17 and 4.84 ppm corresponded to the single -CH_3_ group of lactide and the -CH_2_ of glycolide, respectively. The 4.34 ppm peak represented the PLGA-bound CH_2_ of PEG. The peak around 3.65 ppm was related to another -CH_2_ group in PEG, which due to its proximity to the -CH_3_ peak, appeared as a three-horn. The peak around 1.58 ppm was also related to the -CH_3_ group of lactide. Finally, the 7.29 ppm peak was attributed to the solvent (deuterated dichloromethane). 

The ^1^H-NMR spectrum of PLA-PEG-PLA has been shown in Figure 2, b. The same features mentioned for the chemical groups of PLGA-PEG-PLGA are also applicable for PLA-PEG-PLA.

*Molecular weight determination by GPC*

**Figure 3:**
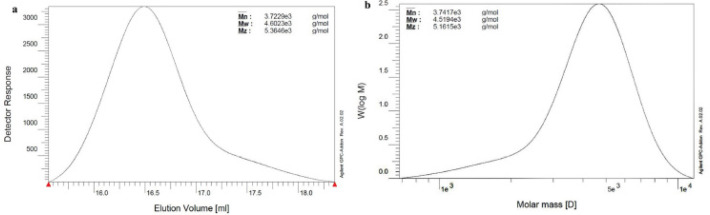
GPC spectra of PLGA-PEG-PLGA (a) and PLA-PEG-PLA (b) triblock copolymers

Figure 3 (a and b) shows the weight** average molecular weight (Mw), number average molecular weight (Mn), and molecular mass distribution of PLGA1500-PEG 1500-PLGA 1500 and PLA 1500-PEG 1500-PLA 1500 triblock copolymers, respectively, as obtained by GPC. Both of the chromatograms show the presence of only one peak, indicating the narrow distribution of molecular weight in both polymers. The constituents of the synthesized copolymers according to GPC and ^1^H-NMR analyses have been represented in Table 1.

***Phase transition temperature***

The sol-to-gel phase transition temperature and precipitation temperature for PLGA-PEG-PLGA and PLA-PEG-PLA triblock copolymers have been shown in Table 2. The PLGA-PEG-PLGA polymer showed a lower precipitation temperature. Also, the phase transition temperature showed an inverse correlation with the concentration of the polymer (Table 2).

***DSC Analysis***

**Figure 4:**
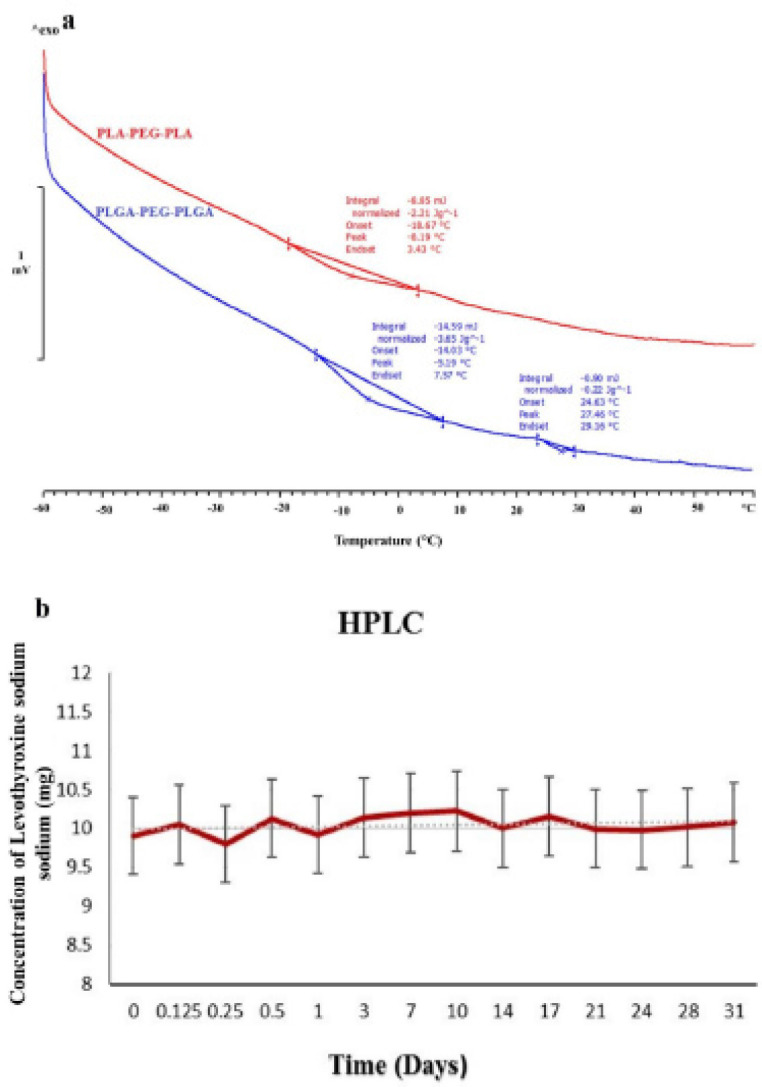
Thermograms of PLGA-PEG-PLGA and PLA-PEG-PLA triblock copolymers (c). Levothyroxine sodium stability based on HPLC analysis during a 31-day period (d)

The thermograms of the fabricated triblock copolymers have been demonstrated in Figure 4a. As can be seen, T_g_ of PLA-PEG-PLA was -8.19 ^°^C, and T_g_ of PLGA-PEG-PLGA was -5.19 ^°^C, and its T_m_ was 27.46 ^°^C.

***Levothyroxine sodium stability***

Levothyroxine sodium stability, according to HPLC, has been indicated in Figure 4b, showing that the drug was stable at 37 ^°^C for one month.

***The syringeability and viscosity of synthesized copolymers***

Both of the triblock copolymers represented good syringeability and could easily pass through a 25G syringe. The viscosity values of PLGA-PEG-PLGA and PLA-PEG-PLA triblock copolymers were obtained at 0.075±0.005 and 0.070±0.004 (Pa.s), respectively, indicating acceptable viscosity for subcutaneous injection. 

***Drug release studies***

**Figure 5:**
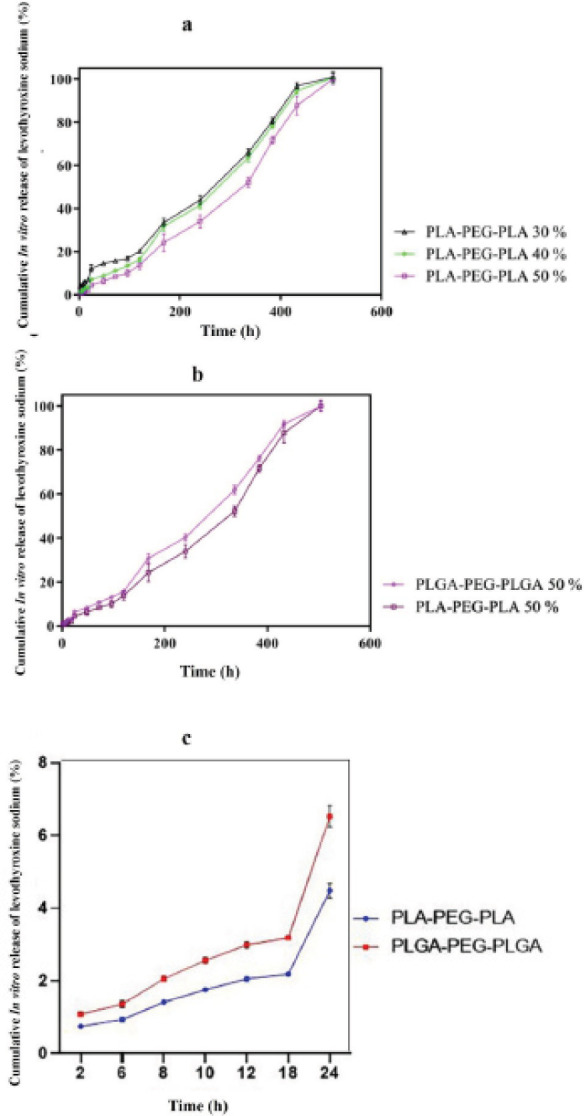
Cumulative release percent of levothyroxine sodium from the formulations containing PLA-PEG-PLA (a). (b) Levothyroxine sodium was released into the NMP solvent from PLGA-PEG-PLGA and PLA-PEG-PLA triblock copolymers containing 50% w/w of the copolymers. (c) Levothyroxine sodium initial burst release into the NMP solvent from PLGA-PEG-PLGA and PLA-PEG-PLA triblock copolymers containing 50% w/w copolymers

The drug release curves of the PLGA-PEG-PLGA were shown in our previous work (Figure 8a) (29), and PLA-PEG-PLA triblock copolymer in the release medium has been demonstrated in Figure 5 a. This experiment was continued for 35 days. The time needed for the complete release of levothyroxine sodium was the same with increasing the copolymer’s concentration, initial burst release gradually slowed and decreased. So, the 50% concentration was chosen as the optimal ratio for both formulations. Finally, the kinetics of drug release, including initial burst release, were assessed for and compared between the copolymers with the 50% w/w concentration. A large amount of the drug was released during the initial moments after the injection (i.e., the first 24 hr), known as “initial burst release”.

Figure 5b shows a comparison of levothyroxine sodium release into the NMP solvent from PLGA-PEG-PLGA and PLA-PEG-PLA triblock copolymers containing 50% w/w concentrations of the polymers, and Figure 5d shows respective drug release in the first 24 hr (i.e., initial burst release). The initial burst release of the triblock containing PLA-PEG-PLA was significantly lower than that of PLGA-PEG-PLGA triblock copolymer, suggesting a smoother drug release by the former. (*P*<0.0001).

**Figure 6:**
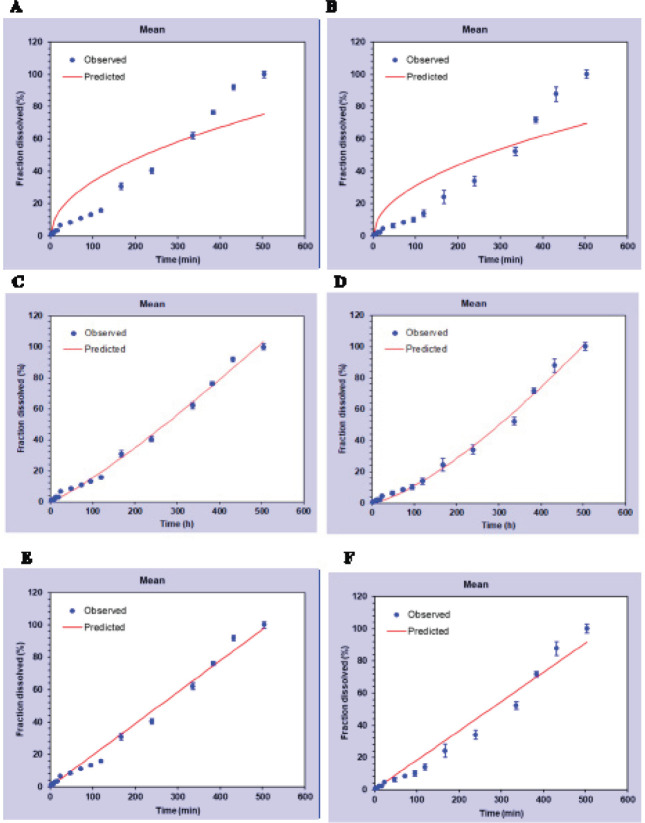
Fitting of release data with different kinetic models. A and B) Higuchi model for drug release from 50% PLGA-PEG-PLGA or PLA-PEG-PLA, C and D) Korsmeyer-Peppas model for drug release from 50% PLGA-PEG-PLGA or PLA-PEG-PLA, E and F) Zero-order model for drug release from 50% PLGA-PEG-PLGA or PLA-PEG-PLA

To evaluate drug release kinetics, levothyroxine release from the formulations containing 50% w/w PLGA-PEG-PLGA and PLA-PEG-PLA was assessed based on the Higuchi, Korsmeyer-Peppas, and zero-order kinetic models (Figure 6), and the respective regression coefficients (R^2^) were compared (Table 3). Also, an “n” index greater than 0.89 in the Korsmeyer-Peppas chart would confirm the zero-degree release model, which was the dominant drug release model from both of the triblock copolymers synthesized in this study.

**Figure 7:**
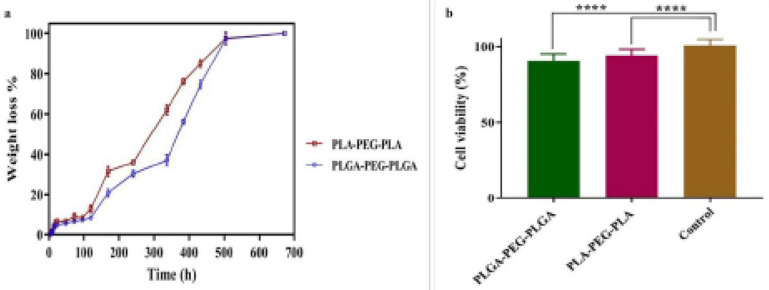
a) Degradation process of PLGA-PEG-PLGA and PLA-PEG-PLA triblock copolymers in deionized water. b) Survival of the skin fibroblast L929 cell line exposed to the formulations containing 50% w/w PLGA-PEG-PLGA and PLA-PEG-PLA

Figure 7a shows the degradation process of the fabricated triblock copolymers, as measured based on the weight loss of the copolymers, showing no detectable changes until 120 hr. At the mentioned time, hydrolytic degradation of the polymers started and then accelerated with time. Within 31 days, PLGA-PEG-PLGA and PLA-PEG-PLA copolymers lost about 87% and 74% of their weights, respectively, indicating a faster degradation rate for PLA-PEG-PLA (*P*<0.001).

***Triblock copolymer effects on the viability of animal cells***

Figure 7b shows the viability percentage of the skin fibroblast L929 cell line exposed to the formulations containing 50% w/w PLGA-PEG-PLGA and PLA-PEG-PLA (90.72±3.251% and 94.13±3.01%, respectively) compared with the control group. Therefore, the formulations did not show considerable cytotoxicity.

## Discussion

Due to its interactions with sodium, calcium, and other minerals, levothyroxine sodium should be used daily and with an empty stomach. In addition, either forgetting to take the medicine in a timely manner or being unaware of the correct way of consumption leads to receiving inappropriate doses of the drug. On the other hand, due to the variable absorption rate (40 to 80%) and relatively low bioavailability (65%) of orally consumed levothyroxine sodium, it is not possible to accurately predict the drug dose entering the blood. Moreover, daily consumption of the drug requires the patient’s full cooperation. To solve these problems, it is required to design a system that can gradually release the drug in a controlled manner within a long period of time even following only one administration (35).

Traditional drug delivery systems, which are based on oral, dermal, and mucosal routes, although suitable for the drugs, are not satisfactory for delivering a wide range of other drugs, such as protein-based and hormonal drugs, especially when long-term administration is required. Other disadvantages of traditional approaches include the fluctuations of drug plasma concentration, drugs’ instability and low solubility, and non-targeted drug delivery. Novel drug delivery systems, on the other hand, allow for targeted long-term drug delivery and drug release, reducing the dosage and side effects of drugs and boosting patient compliance, especially those with chronic diseases (36).

Different formulations of levothyroxine sodium have already been developed to increase its efficiency. Kashanian *et al*. used porous silicon films (as containers) to develop subcutaneously injectable controlled-release (during 60 days) formulations of levothyroxine sodium (37). Other researchers also tried to formulate reverse micelles containing levothyroxine sodium using transdermal films, enabling drug uptake through the skin rather than the gastrointestinal mucosa (38). In another study, Azarbayjani *et al*. developed nanofiber membranes derived from the combination of polyvinyl alcohol and poly N isopropyl acrylamide to produce levothyroxine sodium-containing skin patches (39). Rostami *et al*. also constructed solid lipid nanoparticles (SLN) that released levothyroxine sodium slowly and continuously upon oral administration over the time-examined period (40).

A number of other drugs have been formulated using PLA-PEG-PLA polymers. In a study they used temperature-sensitive PLA-PEG-PLA to deliver doxorubicin to tumors (41). Danafar *et al.* also employed lisinopril-bound PLA-PEG-PLA triblock polymers to fabricate nanoscale micelles (42) carrying atorvastatin (a hydrophobic drug) and lisinopril (a hydrophilic drug), confirming the good applicability of this system for encapsulating and delivering both drugs (43). Researchers successfully employed PLA-PEG-PLA nano-micelles for controlled delivery of naltrexone (44), and another study succeeded in fabricating PLA-PEG-PLA nano-micelle carriers to deliver 5-fluorouracil and paclitaxel to cancer cells (45).

To achieve controlled delivery of levothyroxine sodium, we here used a soluble matrix system, into which the drug was dispersed. By the penetration of an aqueous phase into the system, the matrix swells, allowing the drug to be released from pre-prepared pores. In the end, the polymer is degraded and dissolved. A smart temperature-responsive hydrogel was used, which after being exposed to the body’s aqueous environment and temperature, quickly turned into a gel, providing a gradual and continuous drug release system. We here used the ROP method to fabricate the triblock copolymer system. In this method, lactic acid or glycolic acid monomers are initially opened one by one using a compound containing a polymeric structure (i.e., PEG). The monomers are then joined together sequentially to form a novel polymer. To confirm the successful fabrication of the triblock copolymers, they were characterized using ^1^H-NMR and molecular mass analysis to determine the ratios of lactide to PEG and lactide to glycolide.

In response to temperature, dispersions of polymers in buffer generally take one of the following three phases: sol (completely liquid and fluid), gel (rigid and heavily viscous, remaining at the bottom of an inverted container), and precipitated (completely detached from the solvent, settling down at the bottom of the container). We observed that by increasing the concentration of the copolymer, the sol-gel transition temperature decreased while the precipitation temperature raised. Increasing the temperature and polymer concentration led to micelle formation and facilitated the gelation process. This behavior of some polymers is somehow attributable to their amphiphilic structure (i.e., the simultaneous presence of both hydrophobic and hydrophilic components) (46). In the triblock copolymers fabricated in the present study, PEG was the hydrophilic component, and PLA and PLGA were hydrophobic constituents. When an amphiphilic polymer is dissolved in water, it is separated into tiny unimers surrounded by water molecules. Overall, we observed that the thermal transition points (gelation and precipitation) varied based on the concentration of the triblock copolymers, the ratio of PEG, and the ratio of lactide to glycolide (in PLGA). An increase in the polymer’s concentration and the lactide to glycolide ratio decreases the sol to gel thermal transition point and increases the precipitation temperature, which is explainable by the higher concentration of micelles and their faster accumulation in this condition (47). The PLGA-PEG-PLGA triblock copolymer showed a lower precipitation temperature secondary to the presence of glycolide, allowing water to easily penetrate the 3D network of the gel. The sol-to-gel temperature point is an important feature of a triblock copolymer. In fact, when a copolymer successfully transforms into a gel immediately after being injected into the body, the loaded drug starts to be released gradually and slowly. However, if the copolymer fails to form the gel quickly, a large amount of the drug is abruptly released into the bloodstream, markedly raising its circulating concentrations.

The thermograms of the two fabricated triblock copolymers showed T_g_ values of -8.19 ^°^C and -5.19 ^°^C for PLA-PEG-PLA and PLGA-PEG-PLGA, respectively. The higher temperature of the latter can be due to the presence of glycolide in PLGA, leading to the formation of a more crystalline network compared with PLA. 

Degradation analysis during 31 days showed a higher weight loss (i.e., degradation) for PLGA-PEG-PLGA compared with PLA-PEG-PLA, which can be attributed to the presence of glycolic acid in the former and therefore its more degradability in exposure to humidity (46).

Drug release from the triblock copolymers synthesized into an aqueous medium containing PBS buffer was investigated during the first 24 hr after injection (i.e., the initial burst release) and afterward. In drug delivery systems with a high initial release rate, a large amount of the drug is released during the very first moments, which can cause toxicity. In this study, different ratios of PLGA or PLA to PEG were tried to guarantee the lowest initial burst release. In this study, the initial burst release was determined for six different formulations, among which the PLGA-PEG-PLGA formulation containing 30% hydrogel revealed the highest release rate (17.3±1.2%) within the first 24 hr post-injection. A negative correlation was seen between the concentration of the triblock and the initial burst release rate. Accordingly, the initial burst release at 40% of PLGA-PEG-PLGA was 9.57±2.03% that decreased to 6.52±1.3% at the 50% concentration of this copolymer. Compared with PLGA-PEG-PLGA, the initial burst release of PLA-PEG-PLA was lower, retrieving the values of 12.0±1.7%, 7.06±1.2%, and 4.48±0.7% for 30%, 40%, and 50% concentrations of the triblock, respectively. Overall, the PLGA-PEG-PLGA triblock copolymer delivered a higher initial drug release rate than PLA-PEG-PLA. This observation can be justified by the more hydrophobic nature of PLA compared with PLGA. The hydrophobic nature of PLGA respective to PLA causes this compound, along with its solvent (NMP), to slowly enter the aqueous phase upon being injected into a simulated body environment, blunting initial drug release during the first 24 hr. 

In a study by Azarbayjani *et al*. who employed the two PNIPAM and PVA polymeric systems to deliver levothyroxine sodium via the skin, the initial drug release rate into PBS at 37 ^°^C was 73% for PNIPAM and 48% for PVA during the first 15 min (39). In comparison, the triblock copolymers fabricated in the present study delivered better initial drug release profiles. In another study on levothyroxine sodium-containing skin patches fabricated from aminopropyl triethoxy silane-activated porous silicon, the initial release rate was reported 22.2%, reaching a linear pattern after four days (37). Here, for both PLGA-PEG-PLGA and PLA-PEG-PLA, the formulations containing 50% polymers showed the lowest initial burst release after 35 days.

The time required for each of the triblock copolymers to completely release the drug was assessed during 504 hr (i.e., 21 days). In a study by Rostami *et al. *on the liposomal platforms loaded with levothyroxine sodium, drug release rate over 72 hr was 81% (40), in comparison to which, our synthesized triblock copolymers showed a promising longer and slower release time. Also, in the study of Azarbayjani *et al*., the final drug release rates of two levothyroxine sodium-loaded polymeric systems, PNIPAM and PVA, were 97% and 65%, respectively (39). Moreover, the release of levothyroxine sodium from porous silicone skin patches was reported at 92% after 14 days (37).

In the zero-order drug release kinetic model, the drug is supposed to travel a constant distance by passing through a membrane with a constant thickness to reach the target. In the matrix-based diffusion model, the drug that is closer to the outer surface is initially released, followed by the drug located into deeper segments of the system. In other words, the thickness of the diffusion channel is greater in the central parts of the system (i.e., not following a zero-order release pattern). For the thickness of the drug passage channel to remain constant, the polymer’s degradation should be equivalent to the drug release rate. So, it can be concluded that in matrix systems for zero-order release, drug release should be mainly based on polymer degradation while in the Higuchi model, release occurs mainly via diffusion through matrix. In this study, for both of the synthesized triblock copolymers, the regression coefficient was close to one for zero-order kinetic.

Regarding the Korsmeyer-Peppas model, if the “n” index is ≤0.43, drug release is supposed to follow the penetration model (or Fickian diffusion); if 0.43 ≤ n <0.89, the pattern follows non-Fickian diffusion, and if n ≥ 0.89, drug release is based on polymer degradation (i.e., the zero-order model). In this study, the “n” index for both of the triblock polymers was ≥0.89, confirming the zero-order release pattern and supporting the findings of polymer degradation analysis.

The drug release kinetics of both of the triblock copolymers followed an almost constant and linear pattern except during the first few hours, offering controlled drug-delivery systems with relatively constant drug release rates corresponding to polymer degradation. Although we monitored drug release for 35 days, levothyroxine sodium was completely depleted from all six formulations, independent of the copolymer’s concentration, during 21 days.

**Table 1:** Composition of PLGA-PEG-PLGA and PLA-PEG-PLA triblock copolymers

	**LA/GA** ^a^	**% PEG 1500** ^b^ **(w/w %)**	**M**_n_^c^	**M**_w_^d^	**M**_w_ **/M**_n_ ** (PDI)** ^ e^
**Primary amount**	3.0	30	-	-	-
**PLA 1500-PEG 1500-PLA 1500**	-	28.01	3741.7	4519.4	1.21
**PLGA1500-PEG 1500-PLGA 1500**	2.89	27.14	3722.9	4602.3	1.23

a: LA/GA ratio as determined by ^1^H-NMR, b: PEG percentage as determined by ^1^H-NMR, c: the number average molecular weight by GPC, d: the weight average molecular weight by GPC, e: polydispersity as determined by GPC

**Table 2:** Sol-to-gel phase transition temperature and precipitation temperature of the fabricated PLGA-PEG-PLGA and PLA-PEG-PLA triblock copolymers

Copolymer % w/w concentration	PLGA-PEG-PLGA sol-to-gel transition (23)	PLA-PEG-PLA sol-to-gel transition (23)	PLGA-PEG-PLGA precipitation (23)	PLA-PEG-PLA precipitation (23)
30 ±1.65	28 ±1.65	31±2.33	46±3.15	48 ±1.4
40 ±2. 5	27±2.30	29 ±2.5	52 ±2.9	53±3.25
50 ±2.685	25 ±2.15	28 ±2.9	60±3.8	63 ±2.7

**Table 3:** Regression coefficients based on the fit of levothyroxine sodium release from PLGA-PEG-PLGA and PLA-PEG-PLA triblock copolymers to the Higuchi, Korsmeyer-Peppas, and zero-order models

**Copolymers**	**R** ^2^			**n** Korsmeyer-peppas
	Korsmeyer-peppas F=k*t^n^	Higuchi F= k* t ^1/2^	Zero-order F = k	
PLGA-PEG-PLGA 50 %	0.98	0.81	0.99	1.178
PLA-PEG-PLA 50 %	0.97	0.76	0.98	1.386

## Conclusion

At the concentrations of 30%, 40%, and 50%, PLGA-PEG-PLGA and PLA-PEG-PLA triblock copolymers were in the sol phase at room temperature (25 ^°^C) and under 28 ^°^C, respectively. The triblock copolymers began to transform into a gel, from the above-mentioned temperatures and remain in the gel phase to 60 ^°^C and 63 ^°^C in PLGA-PEG-PLGA and PLA-PEG-PLA triblock copolymers, respectively. It is shown that the two triblocks gel at body temperature (37 ^°^C). The release of the levothyroxine sodium entrapped in these copolymers was slow and continuous. The polymers were biodegradable and biocompatible and had no toxicity against human living cells (L929 skin fibroblast cell line). They were also stable in an anhydrous environment for one month, showing no significant weight loss. After completely releasing the drugs loaded, these polymers are degraded in body fluids and do not need surgery for their removal. The release of levothyroxine sodium from these systems followed a relatively linear pattern with a low initial burst release within one month. The PLGA-PEG-PLGA triblock copolymer delivered a higher initial drug release than PLA-PEG-PLA. This observation can be justified by the more hydrophobic nature of PLA compared with PLGA.

## Authors’ Contributions

JM and FH contributed with conceptualization, supervision, original draft preparation, reviewing, and editing. EK and SNRS helped with writing, reviewing, and editing. BK helped in writing the original draft preparation. HK supervised and helped with writing, reviewing, and editing. FO contributed with conceptualization, supervision, original draft preparation, reviewing, editing, and graphics and picture preparation. 

## Conflicts of Interest

No conflicts of interest were reported by the authors.
